# Enrichment of somatic cancer driver mutations in Alzheimer’s disease microglia and its association with neuroinflammation

**DOI:** 10.1002/alz.084786

**Published:** 2025-01-03

**Authors:** August Yue Huang, Zinan Zhou, Maya Talukdar, Michael B Miller, Brian Chhouk, Liz Enyenihi, Ila Rosen, Edward Stronge, Boxun Zhao, Dachan Kim, Jaejoon Choi, Sattar Khoshkhoo, Junho Kim, Javier Ganz, Kyle J Travaglini, Mariano I Gabitto, Rebecca D Hodge, Eitan S Kaplan, Ed S Lein, Philip L. De Jager, David A. Bennett, Eunjung Alice Lee, Christopher A Walsh

**Affiliations:** ^1^ Boston Children’s Hospital, Boston, MA USA; ^2^ Harvard Medical School, Boston, MA USA; ^3^ Brigham and Women’s Hospital, Boston, MA USA; ^4^ Allen Institute for Brain Science, Seattle, WA USA; ^5^ Columbia University Irving Medical Center, New York, NY USA; ^6^ Rush University Medical Center, Chicago, IL USA

## Abstract

**Background:**

Alzheimer’s disease (AD), an age‐associated neurodegenerative disorder, is characterized by progressive neuronal loss and the accumulation of misfolded proteins such as amyloid‐β and tau. While neuroinflammation, mediated by microglia and brain‐resident macrophages, plays a pivotal role in AD pathogenesis, the intricate interactions among age, genes, and other risk factors remain elusive. Somatic mutations, known to accumulate with age, instigate clonal expansion across diverse cell types, impacting both cancer and non‐cancerous conditions.

**Method:**

Utilizing molecular‐barcoded deep panel sequencing, which enables sensitive detection of somatic mutations with allele fractions as low as 0.1%, we profiled clonal somatic mutations among 149 cancer driver genes in 311 prefrontal cortex samples from AD patients and matched controls. Fluorescence‐activated nuclei sorting and single‐nucleus RNA sequencing were further used to study the cell‐type composition and transcriptomic impact of somatic mutations.

**Result:**

Our study unveiled an elevated occurrence of somatic single‐nucleotide variants and insertions/deletions within cancer driver genes in AD brains. Recurrent somatic mutations, often multiple, were observed in genes associated with clonal hematopoiesis (CH). Remarkably, these somatic mutations were specifically enriched in CSF1R+ microglia and exhibited signals of positive selection, suggesting mutation‐driven microglial clonal expansion (MiCE) in AD brains. Single‐nucleus RNA sequencing of temporal neocortex samples from an additional 62 AD patients and matched controls revealed a nominal increase in mosaic chromosomal alterations (mCAs) associated with CH in AD microglia, with microglia carrying mCA exhibiting upregulated pro‐inflammatory genes, resembling the transcriptomic features of the disease‐associated state in AD.

**Conclusion:**

Our findings indicate that proliferation‐related somatic mutations in microglia are prevalent in normal aging but further enriched in AD, driving MiCE and promoting inflammatory, disease‐related microglial signatures. This study provides crucial insights into microglial clonal dynamics in AD, potentially paving the way for novel approaches to AD diagnosis and therapy.

